# Lingguizhugan decoction alleviates obesity in rats on a high-fat diet through the regulation of lipid metabolism and intestinal microbiota

**DOI:** 10.3389/fmicb.2024.1462173

**Published:** 2024-11-13

**Authors:** Wenjing Huang, Jiuyuan Wang, Zixuan Xiao, Jiayi Lin, Zhoujin Tan, Guixiang Sun

**Affiliations:** College of Chinese Medicine, Hunan University of Chinese Medicine, Changsha, China

**Keywords:** obesity, high-fat diet, Lingguizhugan decoction, intestinal microbiota, short-chain fatty acids

## Abstract

**Background:**

Individuals with obesity often experience elevated blood lipid levels, leading to a chronic low-grade inflammatory state, exacerbating liver oxidative stress, and increasing the risk of various metabolic diseases. Recent evidence suggests that intestinal microbiota and short-chain fatty acids (SCFAs) play crucial roles in the development and progression of obesity. While the mechanisms by which Lingguizhugan decoction (LGZGD) intervenes in obesity by improving lipid metabolism, enhancing insulin sensitivity, and reducing inflammatory responses are well-documented, its potential in intestinal microbiota and SCFAs remains unclear. This study aims to explore the impact of LGZGD on high-fat diet (HFD) induced obesity in rats and its regulatory effects on intestinal microbiota and SCFAs, providing new insights for obesity prevention and treatment.

**Methods:**

Fifty-one male SD rats were randomly divided into groups, with six in the normal control group (NC) receiving a ddH2O treatment and a standard diet. The remaining 45 rats were fed a high-fat diet (HFD) using D12451 feed. After 10 weeks, the rats on the HFD gained 20% more weight than the NC group, confirming the successful modeling of obesity. These rats were then randomly divided into the following groups: ddH2O high-fat diet model group (MC), 20 mg/kg/day Orlistat positive control group (Orlistat), 1.62 g/kg/day low-dose LGZGD group (LGZGL), and 3.24 g/kg/day high-dose LGZGD group (LGZGH) for 8 weeks. We evaluated changes in body weight, serum total cholesterol (TC), total triacylglycerol (TG), low-density lipoprotein cholesterol (LDL), and high-density lipoprotein cholesterol (HDL) levels. Fat and liver tissues were collected for pathological analysis. Intestinal contents were aseptically collected for 16S rRNA gene sequencing and gas chromatography–mass spectrometry (GC–MS) to assess gut microbiota and SCFA levels.

**Results:**

LGZGD reduces body weight, TC, TG, LDL, and HDL levels, significantly reducing hepatic steatosis. Besides, it restored the richness and diversity of gut microbiota, which was reduced by HFD, altering the overall structure. Specifically, LGZGD significantly promoted the growth of *Muribaculaceae* and *Dubosiella* while inhibiting the growth of *Christensenellaceae_R_7_group* and *UCG_005*. It also restricts the production of caproic acid. Correlation analysis indicated positive correlations: *Muribaculaceae* with Butyric acid and Isovaleric acid; *UCG_005* with TC, LDL, and HDL; and *Christensenellaceae_R_7_group* with TC and LDL.

**Conclusion:**

LGZGD increased the abundance of beneficial gut microbiota in HFD-induced obese rats, improved gut microbiota dysbiosis, and inhibited the increase in caproic acid content. These results suggest that LGZGD can mitigate HFD-induced obesity, and its active components warrant further investigation.

## 1 Introduction

Obesity poses a significant threat to global health, exacerbating a range of severe conditions beyond the mere issue of excess weight. It elevates the risk of ailments including type 2 diabetes mellitus (T2DM), non-alcoholic fatty liver disease (NAFLD), hypertension, myocardial infarction, stroke, osteoarthritis, sleep apnea, and multiple forms of cancer (GBD 2015 Obesity Collaborators et al., 2017; Wang et al., 2021). The World Health Organization’s 2016 data reveals that more than 1.9 billion adults globally are overweight, with roughly 650 million falling into the obese category. The prevalence of overweight and obesity in both adults and children, as documented in 2017, persists in escalating, contributing to over 4 million annual deaths linked to overweight or obesity. In China, epidemiological data spanning from 2004 to 2018 shows a staggering increase, with approximately 85 million men and 37 million women now affected by obesity, marking a tripling of cases since 2004.

The primary treatment options for adult obesity are pharmacological and surgical interventions (Perdomo et al., 2023). However, similar to other chronic diseases, obesity requires a long-term, multimodal approach, as different treatments offer varying benefits and risks tailored to individual patient goals (Shi et al., 2024). As our understanding of obesity mechanisms expands, treatment strategies have rapidly evolved, particularly in intestinal microbiota (Geng et al., 2022). The intestinal microbiota, with a bacterial population surpassing the total number of human cells, is often called the “ninth system of the human body” and the “second genome,” functioning as an additional organ. Research has established a causal link between intestinal microbiota and the development of obesity and related metabolic disorders (Bäckhed et al., 2004; Ley et al., 2006; Zhao, 2013). Obese patients typically exhibit an altered intestinal microecology, marked by an increased ratio of Firmicutes to Bacteroidetes, which correlates with body weight and fat accumulation (Liu et al., 2017; Mocanu et al., 2021; Brown et al., 2023). Furthermore, the intestinal microbiota contributes to obesity-related inflammatory responses, oxidative stress, and metabolic dysfunctions associated with intestinal microecological imbalance in the host (de La Serre et al., 2010).

During digestion, the small intestine initially processes and absorbs food. However, not all food components are completely digested and absorbed. Some residues, such as dietary fiber, resistant starch, certain proteins, and fats, along with other substances produced by the host, undergo anaerobic fermentation, converting into intestinal microbiota metabolites. These metabolites mainly include short-chain fatty acids (SCFAs), bile acids (BAs), trimethylamine oxide (TMAO), and compounds containing hydrogen, carbon, and sulfur. SCFAs, in particular, have been linked to obesity development (De Vadder et al., 2014). SCFAs are saturated fatty acids with one to six carbon atoms, also known as volatile fatty acids. Intestinal microbiota produce SCFAs by fermenting undigested carbohydrates, with acetic acid, propionic acid, and butyric acid being the primary components, making up 90 to 95% of the total.

Most Traditional Chinese Medicine (TCM) drugs are orally absorbed, meaning their active ingredients interact with the intestinal microbiota upon entering the intestine. The relationship between TCM and intestinal microbiota has gained significant attention in recent years. Our team has classified and analyzed these interactions and provided a systematic and comprehensive summary of the role of TCM in preventing and treating obesity through the regulation of intestinal microbiota (Huang et al., 2023). Numerous studies have delved into how TCM corrects metabolic disorders by adjusting the composition and function of the intestinal microbiota (Chang et al., 2015; Xu et al., 2015), and some have summarized the mechanisms through which TCM modulates intestinal microbiota in obesity treatment (Zhang et al., 2021). TCM polysaccharide and compound interventions in obesity offer multi-channel, multi-target, and holistic regulation advantages. The mechanisms of TCM intervention in obesity, including improving lipid metabolism, increasing insulin sensitivity, and reducing inflammatory responses, are well-documented. SCFAs have shown inhibitory and ameliorative effects on obesity in animal experiments and some clinical studies, highlighting the importance of TCM in obesity treatment.

**Figure 1:**
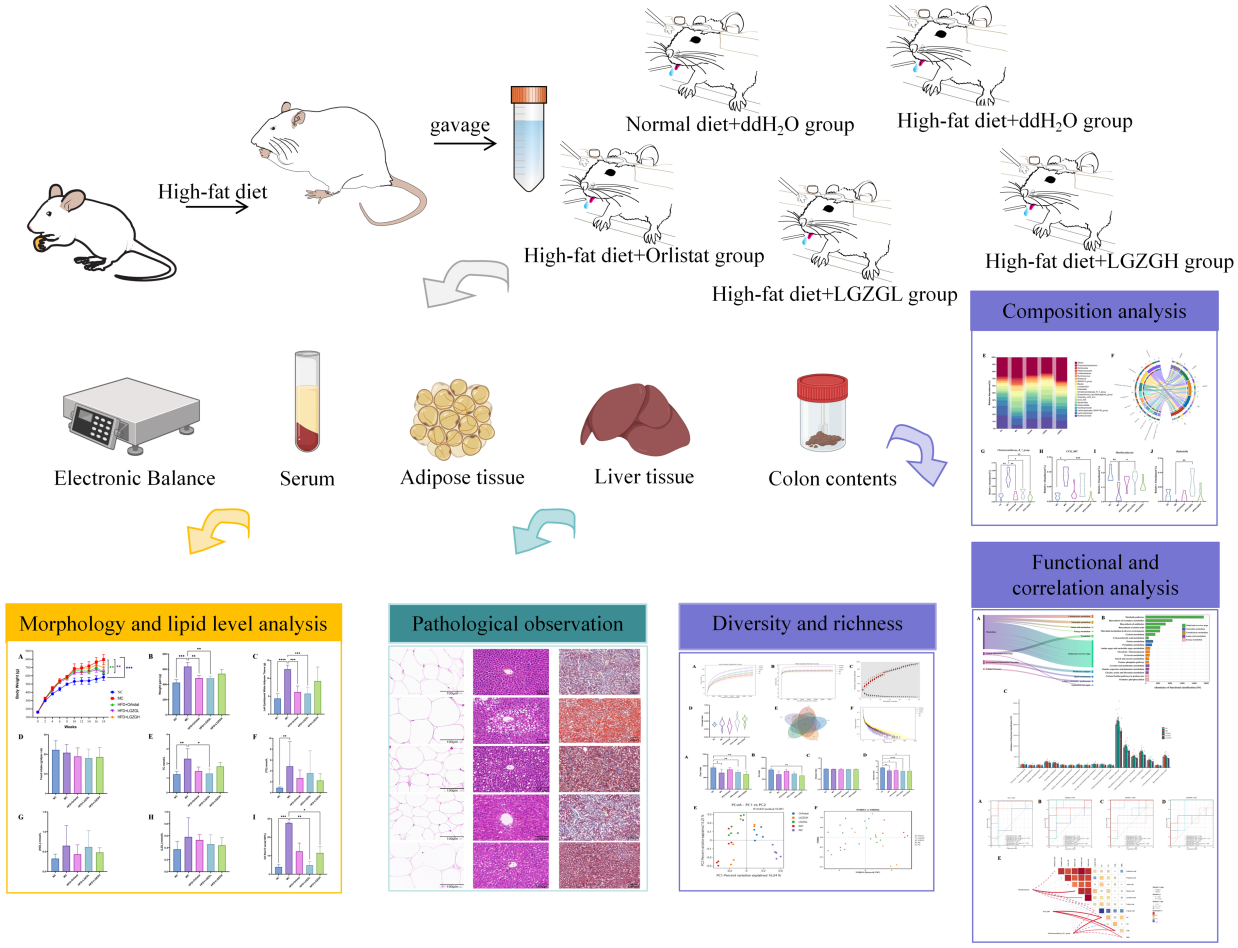
Experimental flow chart.

Professor Guang Ji’s team conducted a multicenter, double-blind, randomized, placebo-controlled clinical trial and found that low-dose LGZGD significantly reduced the insulin resistance index in overweight/obese NAFLD patients compared to placebo. This confirms the efficacy of LGZGD in improving insulin resistance in NAFLD patients with spleen-yang deficiency syndrome, providing evidence-based support for its use in modern chronic disease prevention and treatment (Dai et al., 2022). Based on these findings, we hypothesized that LGZGD improves obesity by regulating lipid metabolism, enhancing the intestinal microbiota structure, and producing short-chain fatty acids. In this study, we investigated the effects of LGZGD on lipid metabolism in an obese rat model and further analyzed its impact on intestinal microbial diversity and short-chain fatty acids content (Figure 1).

## 2 Materials and methods

### 2.1 Preparation of medication and feed

LGZGD was prepared using four Chinese herbal granules as follows: Poria cocos (Schw.) Wolf (Guizhou, batch number NG23041001), *Cinnamomum cassia* Presl (Guangxi, batch number 2023020601, standard: Chinese Pharmacopoeia 2020 Edition Part I), Atractylodes macrocephala (Anhui, batch number TH23030601), and Glycyrrhiza Radix (Inner Mongolia, batch number TH23041704) with a ratio of 12:9:9:6. These four herbs were purchased from the First Affiliated Hospital of Hunan University of Chinese Medicine. According to the original decoction method, the four herbs were soaked in water for 30 min, initially boiled over high heat, and then simmered over low heat for 30 min. The decoction was filtered through three layers of gauze to remove the residue, which was then soaked in water, and the boiling and filtering steps were repeated. The two filtrates were combined and concentrated to a crude drug concentration of 0.6 g/mL, stored in a − 80°C freezer, and reheated to 25–30°C before use.

In this study, the dosing regimen was calculated based on body surface area, incorporating the clinical dosages specified in the Chinese Pharmacopoeia 2020 Edition, the clinical equivalent dose of LGZGD, and previous research indicating that the common oral dose of LGZGD for rats is approximately 3.24 mg/kg (Dang et al., 2020). Taking into account the effects of low (1.62 mg/kg), medium (3.24 mg/kg), and high (6.48 mg/kg) doses of LGZGD on body weight, glucose and lipid metabolism, inflammatory responses, and liver and kidney function in rats, this experiment selected 1.62 mg/kg and 3.24 mg/kg of LGZGD as the intervention doses. The dosage of Orlistat was consistent with that used in previous obesity research (Suleiman et al., 2020). Orlistat capsules (0.12 g per capsule; standard: YBH01682014; Lunan New Time Biotechnology Co., Ltd., Linyi, China) were dissolved in ddH_2_O at a concentration of 20 mg/mL.

The high-fat diet (HFD) was a specially formulated Research Diets-D12451 (fat 45 kcal%, carbohydrates 35 kcal%, protein 20 kcal%), provided by KeAo Xieli Feed Co., Ltd. (Tianjin, China). The regular diet consisted of standard rat maintenance pellet feed provided by the Animal Experiment Center of Hunan University of Chinese Medicine.

### 2.2 Experimental animals

The experimental animals, male 5-week-old SPF-grade SD rats with body weights ranging from 140 to 190 g, were carefully selected from Hunan SLAC Jingda Experimental Animal Co., Ltd., with the production license number SCXK (Xiang) 2019–0004. These animals were chosen for their suitability for the study and were housed at the Experimental Animal Center of Hunan University of Traditional Chinese Medicine, within an SPF-grade barrier system where the environmental conditions were strictly controlled. The experiment adhered to the animal ethics guidelines of Hunan University of Traditional Chinese Medicine, ensuring the ethical treatment of the animals.

### 2.3 Animal model preparation, treatment, and sample collection

SD rats (5 weeks old) were acclimated for 1 week in an SPF-grade rodent barrier before being randomly assigned based on body weight into a control group of 6 rats and a high-fat diet group of 45 rats, with 3 rats per cage. Food and water were provided *ad libitum*, with the control group receiving standard chow and the high-fat diet group receiving Research-Diet D12451 high-fat chow throughout the study. The high-fat diet was stored in a refrigerator and thawed before feeding.

At the end of the 10th week, body weights were measured, and the obesity model was considered established when the body weight of the high-fat diet group exceeded 20% of the control group’s body weight (Kleinert et al., 2018). Qualified models were selected for further treatment: regular diet with ddH_2_O (NC), *n* = 6; HFD with ddH_2_O (MC), *n* = 6; HFD + LGZGL (LGZGL), *n* = 6; HFD + LGZGH (LGZGH), *n* = 6; HFD + Orlistat (Orlistat), *n* = 6. Starting from the 10th week, the LGZGL group received an oral dose of 1.62 g/kg/day of LGZGD, the LGZGH group received 3.24 g/kg/day of LGZGD, and the Orlistat group received 10 mg/kg/day of Orlistat, administered twice daily until the 18th week.

These doses were calculated by converting the equivalent body surface area between animals and humans. Body weight was recorded weekly. At the intervention’s 18th week, all rats were fasted for 12 h, anesthetized with pentobarbital, disinfected with 75% ethanol, and blood was drawn from the abdominal aorta. The whole blood was allowed to stand for 2 h before being centrifuged at 3000 rpm and 4°C for 15 min, after which the serum was collected and stored in a −80°C freezer. Colon contents were collected under sterile conditions on a clean bench, with sequencing samples and short-chain fatty acids detection samples separately placed in 2 mL sterilized centrifuge tubes, immediately frozen in liquid nitrogen, and then transferred to a −80°C ultra-low temperature freezer for storage. The collected samples were sent to Beijing Biomarker Technologies Co., LTD. for subsequent testing. Left epididymal white adipose tissue and liver tissue were collected, weighed, and subjected to histopathological analysis. This animal experiment was approved by the Animal Care and Use Committee of Hunan University of Traditional Chinese Medicine (Animal Ethics Number LL2023030108). All studies were conducted following the recommendations of the National Institutes of Health Laboratory Animal Care and Use Guidelines.

### 2.4 Biochemical index detection

Total cholesterol (TC), total triglyceride (TG), low-density lipoprotein cholesterol (LDL), and high-density lipoprotein cholesterol (HDL) biochemical assay kits were purchased from Nanjing Jiancheng Bioengineering Institute Co., LTD. Following the instructions provided with the kits, absorbance was measured using a MicroplateReader (Shenzhen Huisong Technology, MB-530), standard curves were plotted, and sample concentrations were calculated.

### 2.5 Pathological observation

#### 2.5.1 Hematoxylin and eosin (H&E) staining

Liver and adipose tissue samples were fixed in 4% paraformaldehyde and stored at 4°C overnight, followed by paraffin embedding. Each tissue specimen was then sliced into 3–5 sections with a thickness of 4 μm and stained with H&E. Pathological features were observed using a Leica microscope.

#### 2.5.2 Oil red O staining

The liver samples were fixed in 4% paraformaldehyde, stored at 4°C overnight, dehydrated in 20% sucrose solution, sliced into 5 μm sections with a freezing microtome, and stained according to the conventional pathological process. The amount of lipid deposition was quantified by measuring the proportion of oil red O staining using color thresholding and measuring the red intensity with Image-Pro Plus.

### 2.6 16S rRNA high-throughput sequencing

Samples for this study were sent to Beijing Biomarker Technologies Co., LTD. for testing. Second-generation high-throughput sequencing technology was used to sequence the 16S rRNA V3V4 region. Specific primers (F: ACTCCTACGGGAGGCAGCA and R: GGACTACHVGGGTWTCTAAT) were used for PCR amplification. The amplification products were purified, quantified, and normalized to form libraries for sequencing. After quality control and confirmation that the libraries met standards, sequencing was performed on the Illumina NovaSeq 6,000 platform. The raw image data generated by sequencing were converted into raw sequence reads through base calling and stored in FASTQ format, containing sequence information and corresponding quality information. The raw sequences were quality-controlled to retain high-quality sequences for further analysis.

### 2.7 Short-chain fatty acids content determination

Standard solution preparation: Acetic acid, propionic acid, butyric acid, caproic acid, valeric acid, isobutyric acid, and isovaleric acid pure standards were mixed with water to prepare a 100 mg/mL stock solution and diluted accordingly to prepare a series of working standard solutions. Ethyl was used to mix caproic acid. Internal standard (4-methyl valeric acid) was prepared in ether at 375 μg/mL, and a series of working standard solutions of six acids at 200 μg/mL, 100 μL of 15% phosphoric acid, 20 μL of caproic acid series working standard solution, 20 μL of internal standard, and 260 μL of ether were mixed to prepare a series of standard curve points with specific concentrations set at 0.02, 0.1, 0.5, 2, 10, 25, 50, 100, 250, and 500 μg/mL. The stock solutions were stored at −20°C, and working solutions were freshly prepared. For metabolite extraction, an appropriate amount of sample was taken in a 1.5 mL centrifuge tube, 500 μL of water and 100 mg of glass beads were added, and the mixture was homogenized for 1 min. It was then centrifuged at 12000 rpm and 4°C for 10 min, and 200 μL of the supernatant was taken, added with 100 μL of 15% phosphoric acid, 20 μL of internal standard solution, and 280 μL of ether, homogenized and centrifuged again, and the supernatant was analyzed by chromatography-mass spectrometry.

Chromatography conditions (Hsu et al., 2019; Zhang et al., 2019): Thermo Trace 1,300 gas chromatography system, with Agilent HP-INNOWAX capillary column (30 m × 0.25 mm ID × 0.25 μm); split injection mode, injection volume 1 μL, split ratio 10:1. The injector temperature was set at 250°C; ion source temperature at 300°C; transfer line temperature at 250°C. Temperature program: initial temperature 90°C; then increased at 10°C/min to 120°C; then increased at 5°C/min to 150°C; finally increased at 25°C/min to 250°C and held for 2 min. Helium was used as the carrier gas at a flow rate of 1.0 mL/min.

Mass spectrometry conditions (Hsu et al., 2019; Zhang et al., 2019): Thermo ISQ 7000 mass spectrometer, with electron impact ionization (EI) source, SIM scan mode, and electron energy 70 eV.

### 2.8 Bioinformatics and statistical analysis

Utilizing the DADA2 method (Callahan et al., 2016) within QIIME2 (version 2020.6) (Bolyen et al., 2019), we denoised the data after quality control and set the default threshold for filtering ASVs to 0.005% of all sequenced reads. Subsequently, the SILVA database was employed as a reference, and the Naive Bayesian classifier was used to annotate the taxonomy of the feature sequences. The alpha and beta diversities were demonstrated by Ace, Chao1, Shannon, Simpson indexes, principal coordinates analysis (PCoA), nonmetric multi-dimensional scaling (NMDS) analysis, and analysis of similarities (ANOSIM). Additionally, the depth of 16S rRNA gene sequence of the intestinal microbiota was evaluated using the rarefaction curve, rank abundance curves, and coverage index. Based on these features, taxonomic analysis was conducted to evaluate the distribution of samples across different taxonomic levels from phylum to genus, and results were displayed using community structure plots and chord diagrams. Subsequently, we used Linear Discriminant Analysis Effect Size (LEfSe) to screen key biomarkers (Redinger, 2009). We classified the Kyoto Encyclopedia of Genes and Genomes (KEGG) using PICRUSt2. To evaluate the diagnostic efficiency of the differential genera selected by LEfSe analysis, we constructed the receiver operating characteristic (ROC) curve of each statistically significant differential genus and calculated the area under the curve (AUC). AUC ranges from 0 to 1, with values closer to 1 indicating higher diagnostic efficiency. In addition, to further analyze the correlation between LGZGD and dominant bacteria, serum markers, and SCFAs, we employed the Spearman correlation coefficient and created a correlation heatmap.

Descriptive statistics were expressed as mean ± standard deviation (mean ± SD), and statistical analysis and graphing were performed using IBM SPSS 26.0 software (IBM, Corporation, Armonk, New York, NY, USA) and GraphPad Prism 9.0 software. For normally distributed data, two-group comparisons used unpaired *t*-tests, and multiple-group comparisons used one-way or two-way ANOVA. We performed pairwise comparisons using Tukey’s test. When each mean needed to be compared with a control mean, we chose Dunnett’s test. Multiple group comparisons used Brown-Forsythe and Welch ANOVA tests for normally distributed data with unequal variances. Multiple group comparisons were used for non-normally distributed data, using Kruskal-Wallis tests. When a significant difference was detected, pairwise comparisons were performed using Dunn’s test. The results were considered significant when *p* < 0.05.

## 3 Result

### 3.1 Body weight, food intake, fat mass analysis

**Figure 2:**
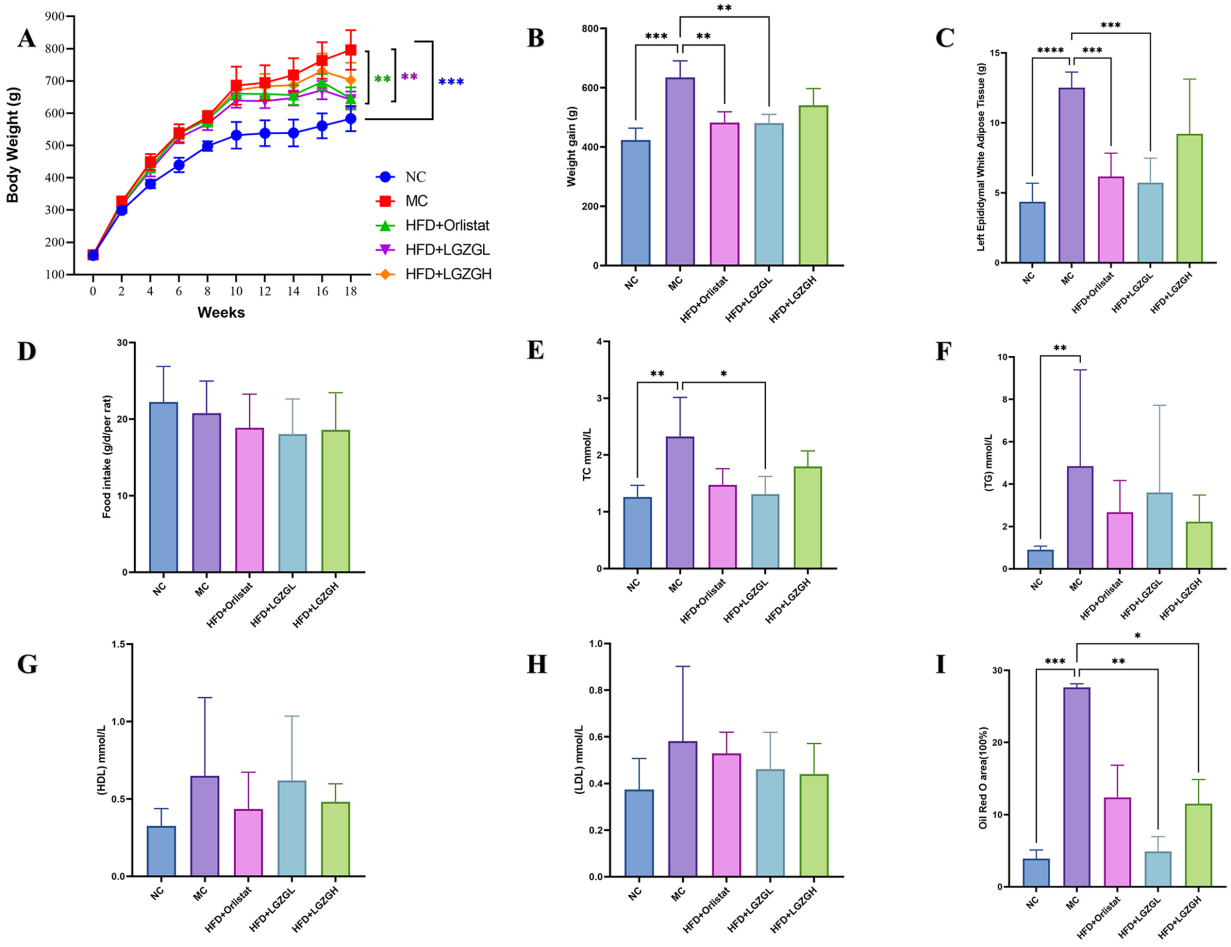
LGZGD alleviated the obesity process in HFD rats. (A) Weekly body weight curves. (B) Weight gain from the 2nd week to the 18th week. (C) Weight of the left epididymal fat. (D) Total food intake during drug intervention. (E) TC. (F) TG. (G) HDL. (H) LDL. (I) Oil Red O staining lipid droplet area ratio. Data were mean ± SD, *n* = 6, *p < 0.05, **p < 0.01, ***p < 0.001, ****p < 0.0001. NC, negative control group; MC, model control group; HFD, high-fat diet; LGZGL, HFD low-dose LGZGD treatment; LGZGH, HFD high-dose LGZGD treatment.

As shown in Figure 2A, after 18 weeks of intervention, the body weight of rats in the MC group increased significantly by 36.5% compared to the blank group (NC) (*p <* 0.001). Compared to the MC group, the body weight of the LGZGL group and the Orlistat group was significantly reduced by 19.38 and 18.97%, respectively (*p <* 0.01). However, the body weight of the LGZGH group decreased by 11.7%, which was not statistically significant. Figure 2B illustrates the body weight growth of rats in each group. The MC group exhibited a 49.8% higher body weight growth compared to the NC group (*p <* 0.05). In comparison to the MC group, the LGZGL and Orlistat groups showed reductions in body weight growth by 24.2 and 23.9% (*p <* 0.01), while the LGZGH group showed an 11.7% decrease.

Regarding epididymal white fat wet weight, the MC group had a significantly higher fat weight (*p <* 0.0001). Compared to the MC group, the fat weight was significantly lower in the LGZGL and Orlistat groups by 54.2 and 50.8%, respectively (*p <* 0.001), whereas it was 26.3% lower in the LGZGH group (Figure 2C). In terms of food intake, there was a decrease in the administered groups compared to both the model and blank groups, but this reduction was not statistically significant (Figure 2D).

### 3.2 Lipid, adipose tissue, and liver histology analysis

As seen in Figures 2E–H, both the LGZGL group and the Orlistat group significantly reduced the levels of TC by 43.48 and 36.56%, respectively, compared to the MC group (*p <* 0.05). Although the treatment groups did not reach statistically significant differences in TG and LDL levels, they exhibited an overall decreasing trend. Meanwhile, HDL levels in the LGZGL group showed an increasing trend.

**Figure 3:**
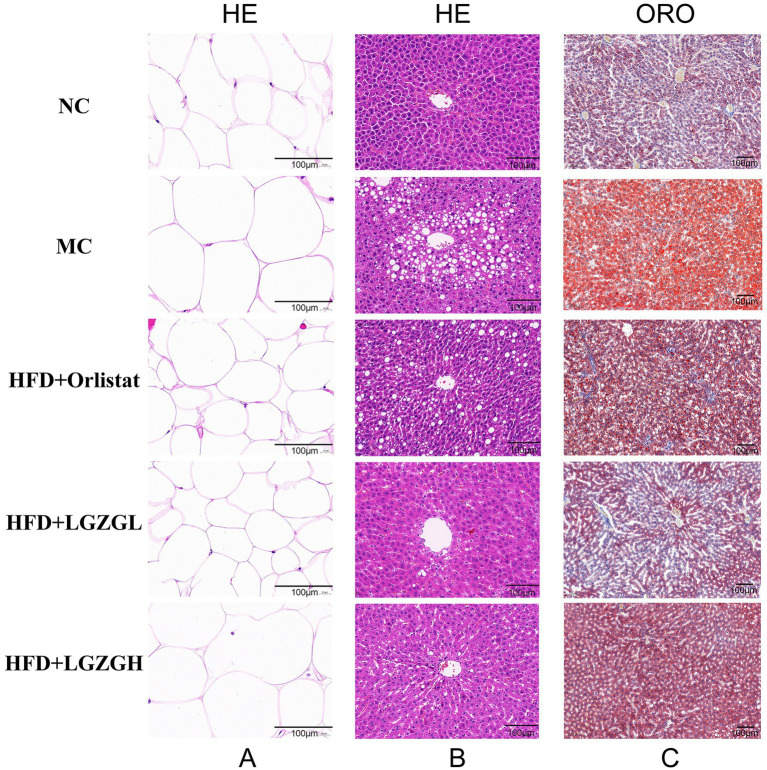
LGZGD alleviate hepatic steatosis in HFD rats. (A) Representative images of epididymal fat stained with H&E (scale bar, 100 μm). (B) Representative images of liver stained with H&E (scale bar, 100 μm). (C) Representative images of liver stained with Oil Red (scale bar, 100 μm).

Figure 3A presents the results of HE staining of white fat in the left epididymis. The diameter of adipocytes in the MC group was significantly enlarged, with only 5 adipocytes observed under 400× magnification, compared to 26 adipocytes in the Orlistat group and 31 adipocytes in the LGZGL group. The cell sizes in the LGZGL and Orlistat groups were very similar to those in the NC group.

Compared with the normal group, hepatic steatosis was evident in the model group, characterized by significant fat accumulation and infiltration, as well as blurred cell borders (Figure 3B). These phenomena were alleviated in all treatment groups, with the LGZGD group showing a healthy liver with relatively intact cell borders, well-preserved cytoplasm, and visible nuclei. Further quantitative analysis of oil red O staining showed that the MC group had significant red staining of lipid droplets (Figure 3C). The LGZGL and LGZGH groups significantly reduced the ratio of hepatic lipid droplet area (*p <* 0.05), while the Orlistat group showed a tendency to reduce it (*p* > 0.05) (Figure 2I). These results are consistent with previous studies indicating that LGZGD intervention is effective in preventing and ameliorating hepatic steatosis.

These results suggest that LGZGD can effectively prevent obesity and lipid metabolism disorders in HFD rats. The inhibitory effect of the LGZGL group on weight gain in HFD obese rats was better than that of the LGZGH group, and its effect was comparable to that of the Orlistat group.

### 3.3 Quality assessment of microbiota sequencing data from rat colon contents and analysis of OTUs

**Figure 4:**
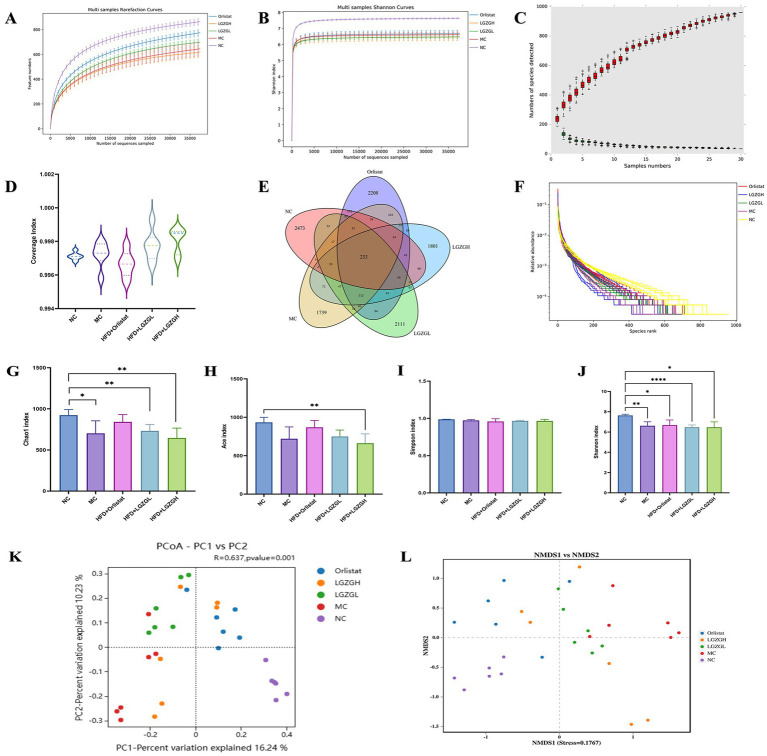
Sequencing data quality assessment of intestinal contents microbiota and OTU number and diversity of intestinal contents microbiota. (A) Dilution curve of Chao1. (B) Dilution curve of Shannon. (C) Species accumulation curve. (D) Sample coverage index. (E) Venn diagram. (F) Rank abundance distribution curve. The longer the folded line, the greater the number of OTUs in the sample. The gentler the curve, the better the uniformity of the community; the steeper the curve, the lower the homogeneity of the community. (G) Chao1 index. (H) Ace index. (I) Simpson index. (J) Shannon index. (K) PCOA analysis. (L) NMDS analysis. Data were mean ± SD, *n* = 6, *p < 0.05, **p < 0.01, ***p < 0.001, ****p < 0.0001.

After quality control, 2,907,953 raw sequencing data points were obtained. The dilution curve can be used to assess whether the sequencing depth is sufficient to cover all taxa and thus reflect the species diversity in the samples. As the number of OTUs sequenced in each group increases, the curve reaches a plateau when the amount of sequences per sample reaches 5,000, indicating that the microbial biomass detected in each sample is nearing saturation (Figures 4A,B). This suggests that the current sequencing depth is sufficient to reflect the microbial diversity in this batch of samples. As more samples are added, the increase in the number of OTUs slows down, and the curve flattens out. The total number of OTUs remains constant as the curve levels off, indicating that the sample size in this study is adequate (Figure 4C). Therefore, we hypothesize that a reasonable sequencing depth was used, and the amount of data sequenced from the samples sufficiently represents the true microbial community in each sample, allowing for accurate microbial diversity analysis. Coverage values were also calculated, with higher values indicating a higher probability that a species was sequenced in the sample and a lower probability that it was not. The coverage values for this sequencing were all greater than 0.996, representing the true microbial situation in the samples (Figure 4D).

Further observing the number of OTUs, the NC group, MC group, Orlistat group, LGZGL group, and LGZGH group had 3,327, 2,672, 3,347, 3,065, and 2,788 OTUs, respectively, with 233 OTUs shared among the five groups (Figure 4E). The rank abundance distribution curve, drawn based on abundance log2 values (Figure 4F), showed that the number of OTUs in each intervention group was higher than in the MC group. This finding aligns with the Venn diagram results, suggesting that LGZGD may effectively mitigate the reduction in intestinal microbiota caused by obesity.

### 3.4 Analysis of the diversity of rat colon contents microbiota

In this study, the Chao1, Ace, Shannon, and Simpson indices were used to measure Alpha diversity, which reflects the species richness and diversity of individual samples. The Chao1 and Ace indices measure species richness, i.e., the number of species, while the Shannon and Simpson indices measure species diversity, which is influenced by both species richness and community evenness within the sample community (Figures 4G–J). For the same species richness, greater evenness in the community results in higher diversity, as indicated by higher Shannon and Simpson indices, thus reflecting greater species diversity in the sample (Grice and Segre, 2011). In this study, the Chao1, Ace, and Simpson indices were slightly elevated in the Orlistat and LGZGL groups compared to the MC group. All four indices showed a downward trend compared to the NC group, indicating that the LGZGL intervention had some regulatory effect on the diversity of the intestinal microbiota.

Beta diversity measures differences in population distribution across different environmental communities and assesses the similarity between different sample communities. This experiment used Principal Coordinates Analysis (PCoA) and Analysis of Similarity (ANOSIM) based on the Bray-Curtis algorithm to further demonstrate species diversity differences and similarities among samples. As shown in Figure 4K, the samples from the MC group were located in the first and third quadrants, while the NC group was separated from the MC group. The LGZGD and Orlistat groups were situated between the NC and MC groups. The ANOSIM results showed significant differences between all groups (*R* = 0.637, *p <* 0.001). The NMDS results were consistent with the PCoA analysis (Figure 4L), demonstrating that the HFD reduced the richness and diversity of the intestinal microbiota and altered its overall structure. The intervention groups improved the intestinal microbiota diversity of HFD rats to varying degrees.

### 3.5 Analysis of the microbiota structure of rat colon contents

**Figure 5:**
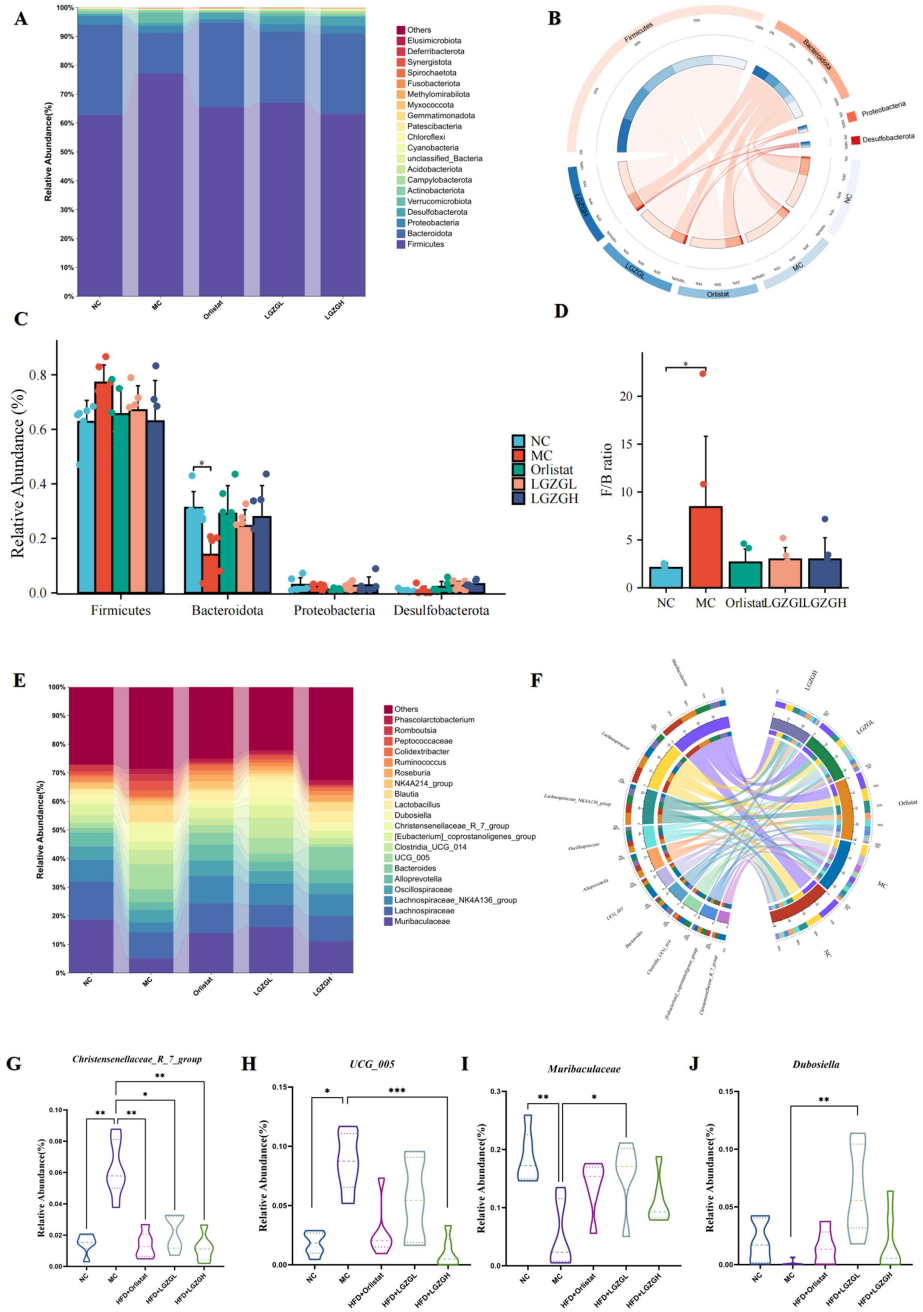
Dominant bacteria and differential bacteria of intestinal contents microbiota. Data were mean ± SD, *n* = 6,*p < 0.05, **p < 0.01, ***p < 0.01. (A) Intestinal contents microbiota composition in the phylum level. (B) Chord diagram of the phylum level. (C) Multi-species comparison at the phylum level. (D) Comparison of Firmicutes/Bacteroidetes Ratio. (E) Intestinal contents microbiota composition in the genus level. (F) Chord diagram of the genus level. (G–J) Multi-species comparison at the genus level.

Figure 5A presents the relative abundance of the top 20 intestinal microbiota at the phylum level. Among these, Firmicutes, Bacteroidota, Proteobacterota, and Desulfobacterota were the dominant phyla across the five groups. Based on species with relative abundance greater than 1%, we used a chord diagram to summarize the dominant phyla in terms of abundance (Figure 5B). Statistical analysis of these dominant phyla revealed that the differences in the levels of Firmicutes were decreased (*p >* 0.05) (Figures 5C,D). Bacteroidota were significantly lower (*p <* 0.05), and the F/B ratio was significantly higher (*p <* 0.05) in the MC group compared to the NC group. However, the microbiota disturbance due to HFD was alleviated with the increase in LGZGD dose. Specifically, LGZGD inhibited the increase of Firmicutes and promoted the growth of Bacteroidota.

Figure 5E shows the relative abundance of the top 20 intestinal microbiota at the genus level. Based on the top 10 species ranked by relative abundance, we applied a chord diagram to summarize the dominant bacterial genus with abundance greater than 1% (Figure 5F). Statistical analysis of these genus (Figures 5G–J) revealed significantly higher abundances of *Christensenellaceae_R_7_group* and *UCG_005* in the MC group compared to the NC group (*p <* 0.01; *p <* 0.05). The abundance of *Christensenellaceae_R_7_group* and *UCG_005* was significantly reduced in the LGZGH group compared to the MC group (*p <* 0.01; *p <* 0.001), and the abundance of *Christensenellaceae_R_7_group* was significantly reduced in the LGZGL group (*p <* 0.05). Conversely, there was a significant reduction in the number of *Muribaculaceae* in the MC group compared to the NC group (*p <* 0.01), while the numbers of *Muribaculaceae* and *Dubosiella* were significantly higher in the LGZGL group compared to the MC group (*p <* 0.05; *p <* 0.01).

**Figure 6:**
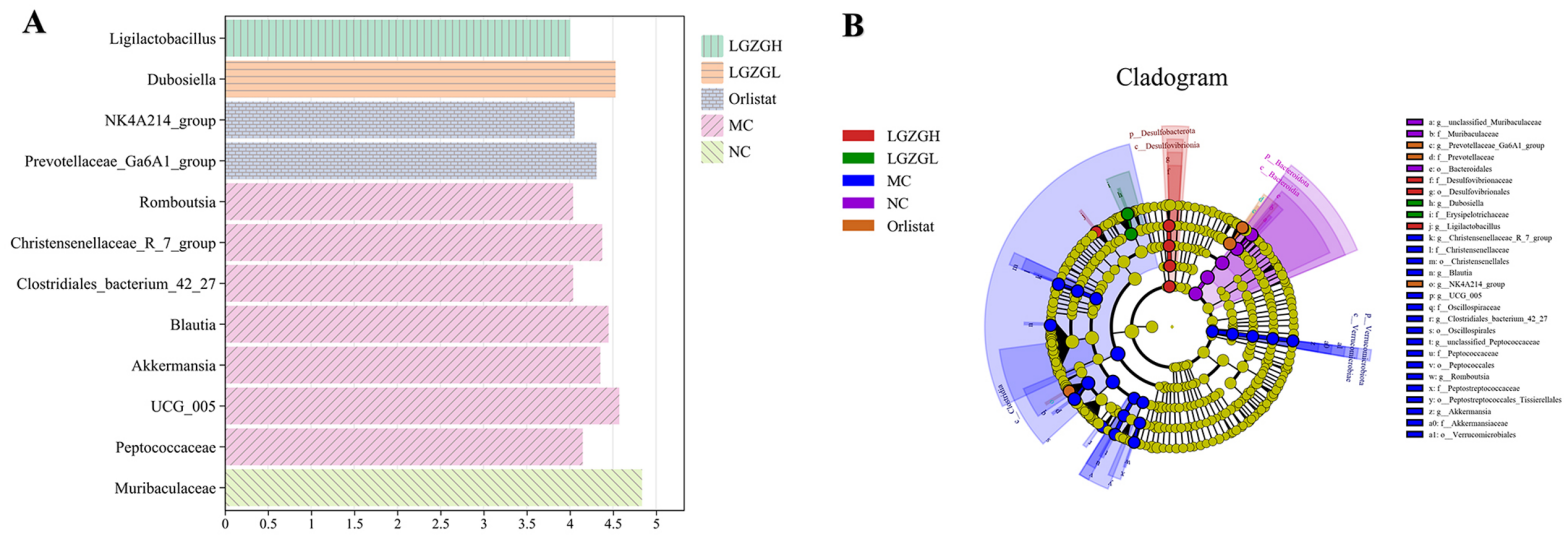
LEfSe analysis. (A) Histogram plots of LDA value distribution of biomarkers between NC, MC, Orlistat, LGZGL and LGZGH groups, listing the LDA score threshold > 4, indicating higher relative abundance of the corresponding groups. (B) Evolutionary Branch Diagram. The evolutionary branch diagram features concentric circles radiating from the center outward, representing taxonomic levels from phylum to genus. Each small circle at different taxonomic levels symbolizes a classification at that level, with the diameter of the circle proportional to the relative abundance. The coloring principle is to uniformly color species without significant differences in yellow, while other distinct species are colored according to the group in which they have the highest abundance. Different colors indicate different groups, and nodes of different colors represent microbial communities that play a significant role in the group represented by that color.

Thus, the LGZGH intervention inhibited the growth of dominant bacteria such as *Christensenellaceae_R_7_group* and *UCG_005*, while LGZGL promoted the growth of *Muribaculaceae* and *Dubosiella*. The results of LEfSe analysis showed that the NC, MC, Orlistat, LGZGL, and LGZGH groups had 1, 7, 2, 1, and 1 dominant taxa, respectively (Figure 6A). Among them, *Christensenellaceae_R_7_group* and *UCG_005* were significantly more abundant in the MC group. The LGZGL group was enriched with *Dubosiella*, while LGZGH was characterized by a higher increase in the number of *Ligilactobacillus*. Differential bacteria significantly varied among the three groups across different taxonomic levels (Figure 6B).

### 3.6 Prediction of PICRUSt2 function in the microbiota of rat colon contents

**Figure 7:**
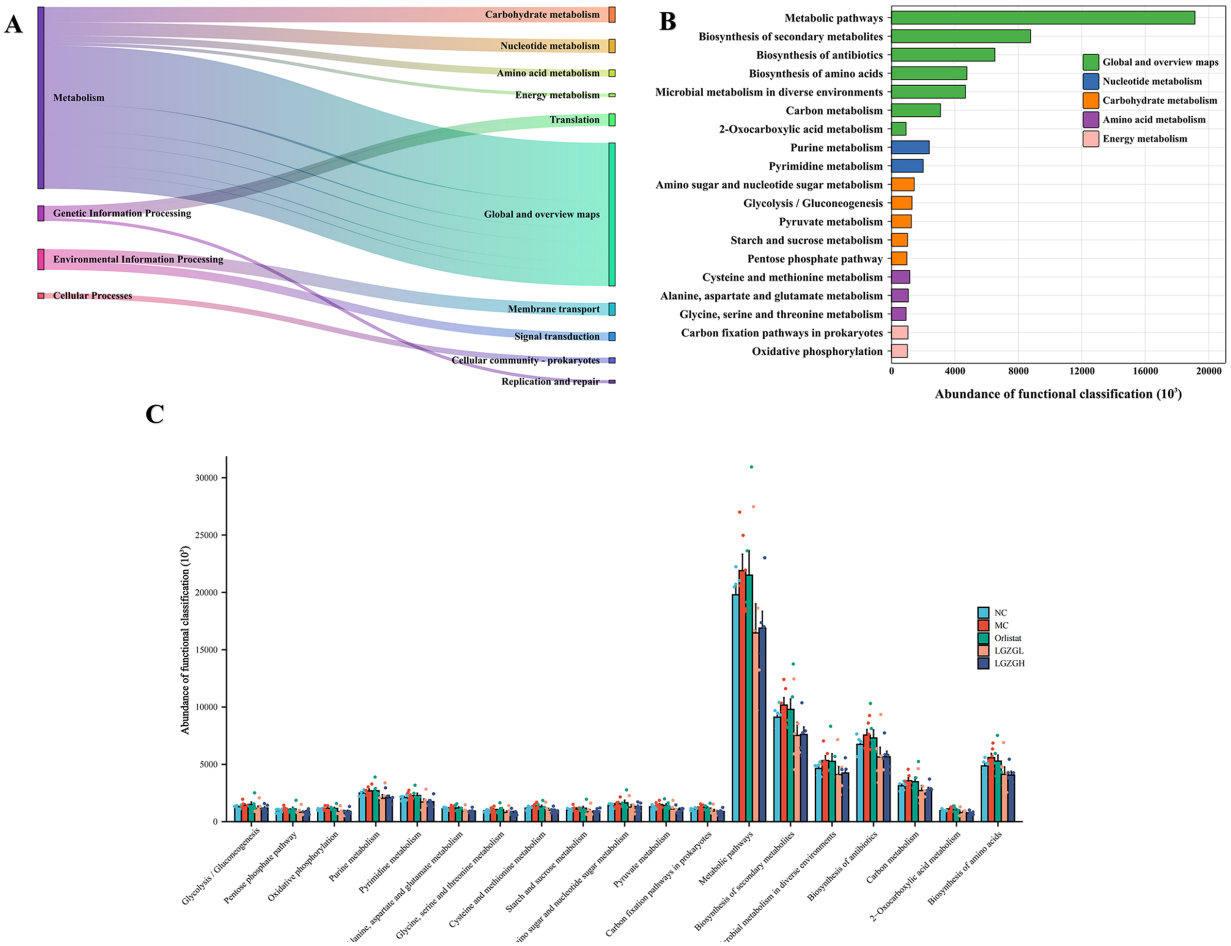
PICRUSt2 analysis of intestinal microbiota; (A) Kyoto Encyclopedia of Genes and Genomes (KEGG) functional categories (levels 1 and 2). (B) Metabolic histogram (levels 2 and 3). (C) Comparisons between the groups for each KEGG functional categories (level 3).

**Figure 8:**
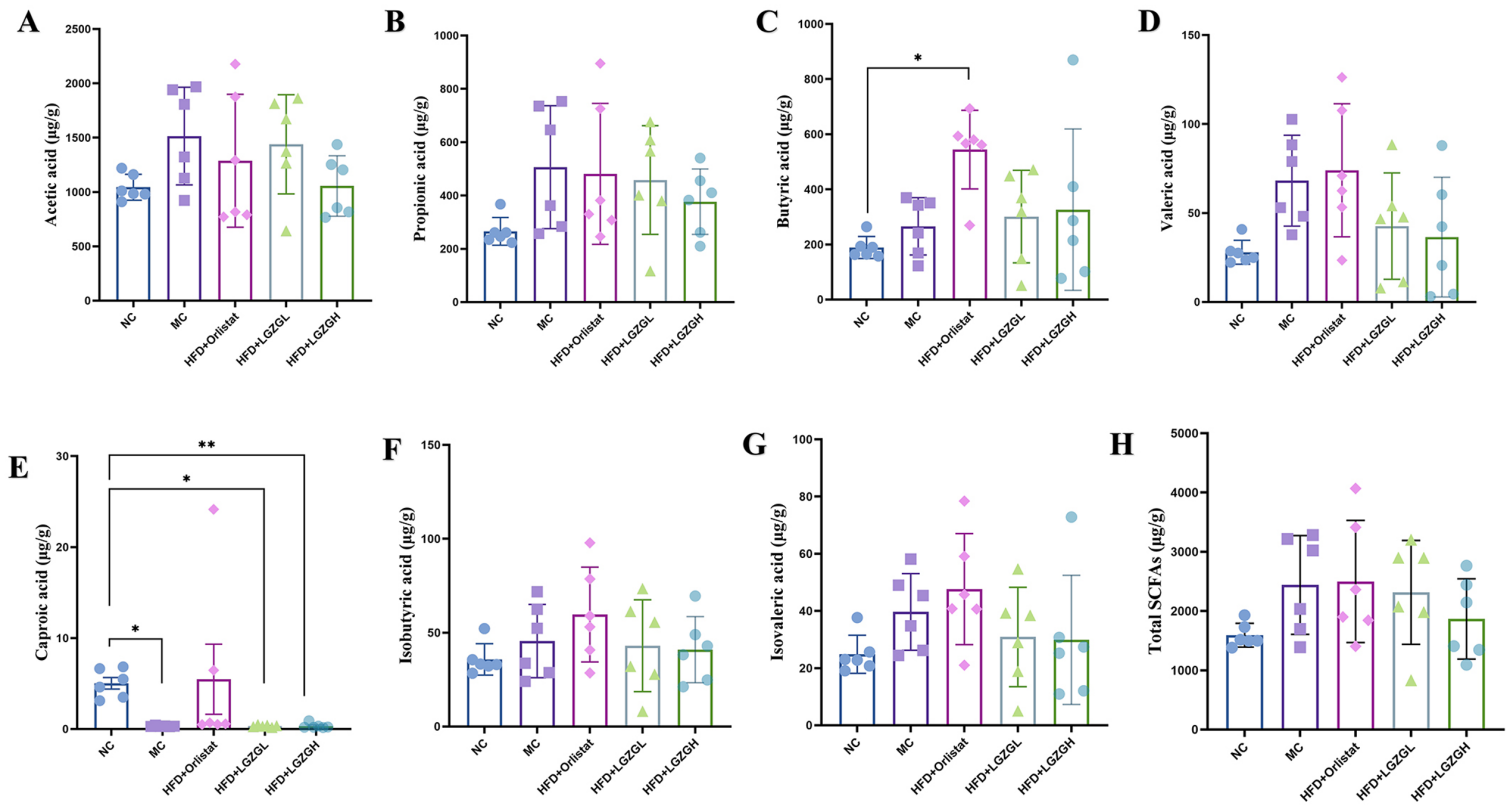
Short-chain fatty acids of rats with a high-fat diet intervened by Lingguizhugan decoction and Orlistat. Levels of short-chain fatty acids in rats. Parts **(A-H)** represent different analytes measured. Data are represented as mean ± SD, *n* = 6, *p < 0.05, **p < 0.01.

The functions of the intestinal microbiota were broadly categorized into 6 classes, with 46 subfunctional categories at the second level, of which the median was >1020654.889 with 21 categories (Figure 7A). Metabolic functions were more abundant with 152 categories, of which the median was >921081.6806 with 19 categories (Figure 7B). As shown in Figure 8B, there was a significant impact on global and overview maps, nucleotide metabolism, carbohydrate metabolism, amino acid metabolism, and energy metabolism. Further analysis revealed significant differences in oxidative phosphorylation, alanine, aspartate and glutamate metabolism, pyruvate metabolism, and metabolic pathways in prokaryotes (Figure 7C). Metabolic pathways were increased in the MC group compared to the NC group. After drug intervention, metabolic activity tended to decrease in all treatment groups, with the most pronounced reduction observed in the LGZGL group, followed by the LGZGH group, and finally the Orlistat group.

### 3.7 Analysis of SCFAs in rat colon contents

Compared with the NC group, the levels of acetic acid, propionic acid, butyric acid, valeric acid, isobutyric acid, and isovaleric acid were elevated in the MC group of rats (*p* > 0.05), while the content of caproic acid was decreased (*p* < 0.05). In the LGZGL and LGZGH groups, levels of acetic acid, propionic acid, valeric acid, isobutyric acid, and isovaleric acid were decreased compared to the MC group (*p* > 0.05), while butyric acid showed an increasing trend (*p >* 0.05). Unlike the LGZGD group, the Orlistat group exhibited increased levels of valeric acid, caproic acid, isobutyric acid, and isovaleric acid compared to the MC group (*p* > 0.05) (Figures 8A–H). These results indicate that the LGZGD intervention inhibited the production of caproic acid in the intestinal contents of obese rats.

### 3.8 Correlation analysis between the characteristic microbiota of rat colon contents and blood lipids and SCFAs

**Figure 9:**
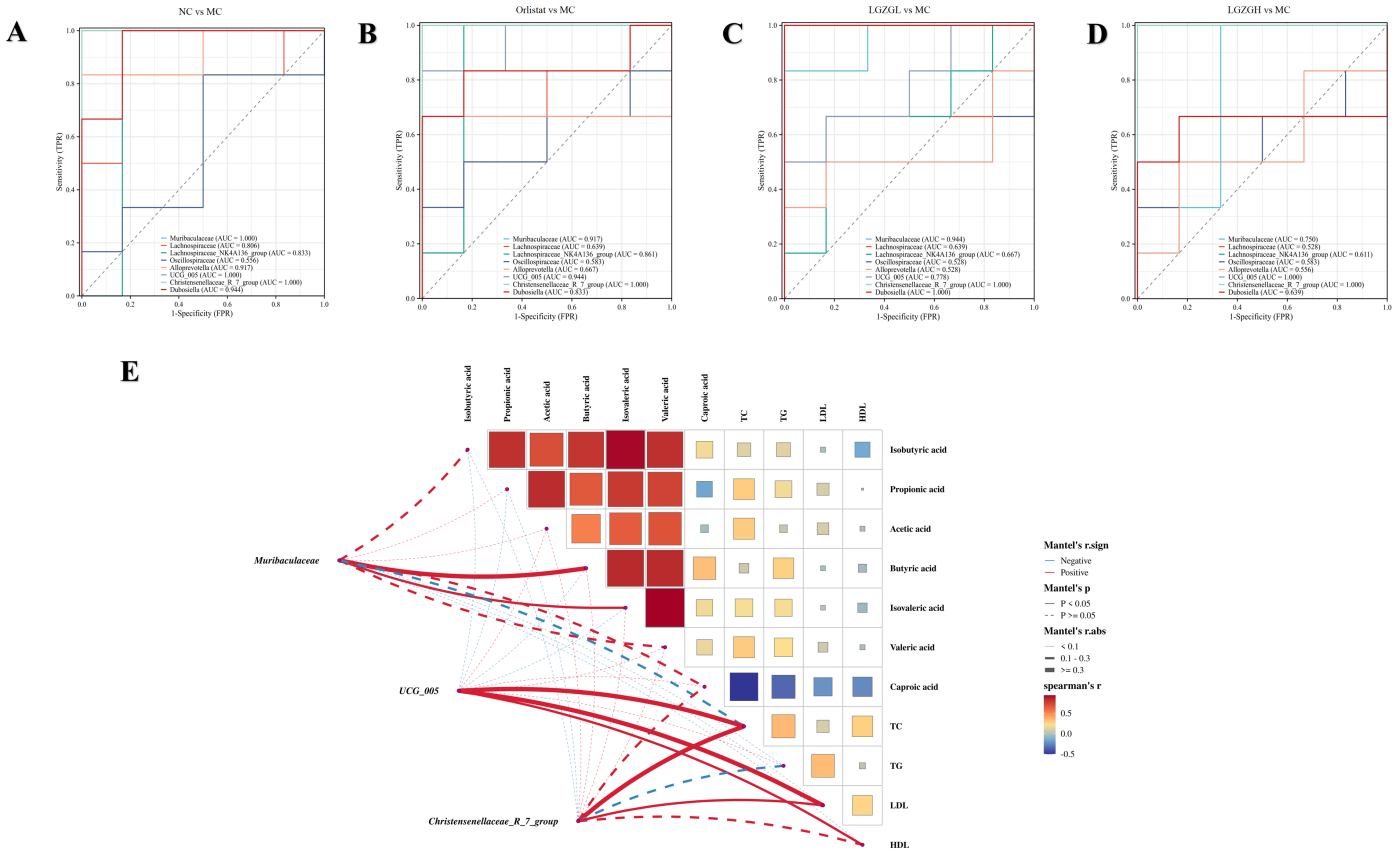
The characteristics of gut microbiota in rats. (A) ROC at the genus level (the NC group vs. the MC group). (B) ROC at the genus level (the Orlistat group vs. the MC group). (C) ROC at the genus level (the LGZGL group vs. the MC group). (D) ROC at the genus level (the LGZGH group vs. the MC group). (E) Association network Heatmap: association network between *Muribaculaceae*, *Christensenellaceae_R_7_group*, *UCG_005*, SCFAs and lipid metabolism. The color spectrum in the legend indicates correlation coefficient values, with red denoting a positive correlation and blue a negative correlation. Solid lines represent statistically significant correlations (*p* < 0.05), while dashed lines indicate non-significant correlations (*p* > 0.05). The lines’ thickness signifies the strength of correlation.

To further investigate the impact of varying doses of LGZGD intervention on the correlation between rat colon microbiota and environmental factors, we employed the Spearman method to analyze the top 10 characteristic genera in each group in relation to lipid levels and SCFAs. To assess the predictive accuracy of these characteristic genera, we evaluated the diagnostic accuracy and the co-evaluation of characteristic microbiota between different groups using an AUC threshold of 0.8. Characteristic microbiota with an AUC greater than 0.8 was considered significant, indicating distinct characteristics between the groups. From the ROC plots (Figures 9A–D), it was observed that the AUC for *Christensenellaceae_R_7_group* in each group was greater than 0.8, and for *Muribaculaceae, UCG_005*, the AUC was greater than 0.75. These genera could be identified as bacteria with a diagnostic effect.

Further research into the relationship between characteristic bacterial genera, SCFAs, and lipid metabolism revealed the following positive correlations: *Muribaculaceae* with Butyric acid and Isovaleric acid; *UCG_005* with TC, LDL, and HDL; and *Christensenellaceae_R_7_group* with TC and LDL. Based on these correlation analyses, it is inferred that the interactions between *Muribaculaceae*, *UCG_005*, and *Christensenellaceae_R_7_group* with the abovementioned factors may constitute the primary mechanism through which LGZGD ameliorates obesity induced by a high-fat diet in rats (Figure 9E).

## 4 Discussion

Adipose tissue and liver are the major endocrine organs involved in the development of obesity. Obesity-induced energy imbalance leads to adipocyte hypertrophy and hepatic lipid deposition (Huffman and Barzilai, 2009; Redinger, 2009; Dushay et al., 2010). In our 18-week study, the results showed that LGZGD effectively inhibited HFD-induced obesity, as evidenced by the tendency of body weight, fat weight, and lipid levels to decrease in each treatment group. There was no significant difference between the LGZGD group and the Orlistat group, indicating that LGZGD had similar effects on reducing obesity in rats. Notably, LGZGL had a better lipid-lowering effect than LGZGH. Subsequent analysis of fat and liver pathology showed a similar trend.

In recent years, the exploration of Chinese medicine research on intestinal microecology and obesity has deepened, continuously updating our understanding of the relationship between intestinal microecology and Chinese medicine. The vast majority of traditional Chinese medicines are administered orally and first contact the gastrointestinal tract, where the active ingredients interact with the intestinal microbiota. Several studies have systematically reviewed and analyzed these interactions, comprehensively summarizing the role of TCM in colony modulation for the treatment of obesity and its significance (Yue et al., 2019; Cui et al., 2023; Feng et al., 2023). This research primarily focuses on two aspects: the interaction between herbal medicines and intestinal microorganisms and the effect of secondary metabolites produced by herbal medicines in the intestines on the host.

In this experiment, we analyzed the intestinal contents microbiota of rats before and after modeling and before and after drug administration using bioinformatics technology. The reliability of the downstream data was confirmed, ensuring that the sample size and homogeneity of the random sampling satisfied the experimental design and provided quality assurance for the subsequent analysis results. We found that LGZGD helped ameliorate the negative effects of HFD, including overweight, hyperlipidemia, hepatic steatosis, and dysfunction of intestinal microbiota.

In terms of diversity results, this experiment analyzed Alpha diversity, Beta diversity, and the number of OTUs. It was found that HFD modeling significantly altered the diversity of intestinal contents microbiota and community structure in rats, which gradually tended to be restored after treatment with LGZGD and Orlistat.

To further analyze the differences in community structure of each group, we examined the dominant phyla and genera that ranked in the top 10 in relative abundance and had a relative abundance ratio higher than 1%. The human intestinal microbiota at the phylum level is usually dominated by Firmicutes and Bacteroidota, accounting for 80–90%. The F/B ratio can be used as a marker for ecological dysbiosis of the intestinal microbiota, with obese patients showing an increased proportion of Firmicutes and a decreased proportion of Bacteroidota (Zhao, 2013; Petakh et al., 2023). In this experiment, after HFD modeling, the relative abundance of Firmicutes in the MC group showed an increasing trend, while Bacteroidota significantly decreased (*p* < 0.05), and the F/B ratio significantly increased (*p* < 0.05). However, the LGZGD intervention inhibited the growth of Firmicutes, increased the relative abundance of Bacteroidota in rats, and restored their F/B ratio to normal levels, similar to the Orlistat group.

We analyzed the top 20 dominant genera with relative abundance rankings and ratios higher than 1% and found that HFD significantly promoted the growth of *Christensenellaceae_R_7_group* and *UCG_005*, while inhibiting the growth of *Muribaculaceae* and *Dubosiella* (*p* < 0.01; *p* < 0.05); (*p* < 0.01; *p >* 0.05). This suggests that the aforementioned genera play a role in the development of obesity, either negatively or positively. Post-intervention with LGZGD, *Christensenellaceae_R_7_group* and *UCG_005* were significantly down-regulated, with this effect being dose-dependent. Notably, only the LGZGL group significantly promoted the growth of *Muribaculaceae* and *Dubosiella* (*p* < 0.01; *p* < 0.05).

*Christensenellaceae_R_7_group* belongs to Firmicutes, whose increase may relate to enhanced calorie extraction from food, fat deposition, and adipogenesis (Li et al., 2023). It has been shown that *UCG_005* is dominant in the NASH group, while *Muribaculaceae* is significantly reduced in the HDF diet group, which aligns with our findings (Lei et al., 2022; Wu et al., 2022). Our results suggest that LGZGD intake leads to structural changes in the dominant genera, reducing harmful bacteria and increasing the abundance of probiotics in the intestinal microbiota of obese rats. Thus, LGZGD can be considered a prebiotic to prevent obesity and hyperlipidemia.

Line Discriminant Analysis Effect Size (LEfSe) analysis revealed the microbial markers for each group, suggesting that *Ligilactobacillus* and *Dubosiella* might play crucial roles as biomarkers in the treatment of obesity with LGZGH and LGZGL, respectively. *Dubosiella* has been shown to ameliorate obesity (Ai et al., 2022), specifically regulated by FGF21, significantly improving liver function and reducing lipid accumulation in mice with NAFLD (Ye et al., 2023). *Dubosiella* produces short-chain fatty acids (especially propionic acid) and lysine (L-Lys), modulating colonic Tregs/lipids, inhibiting colonic inflammatory responses, and ameliorating mucosal barrier damage. The increased abundance of *Dubosiella* in the LGZGD group suggests that LGZGD may improve obesity through this bacterial genus.

Metabolic function was identified as the main functional category affecting LGZGD treatment of obesity through PICRUSt2 function prediction. KEGG results indicated that different doses of LGZGD and Orlistat mainly impacted intestinal microbiota functions such as oxidative phosphorylation, alanine, aspartate and glutamate metabolism, pyruvate metabolism, and carbon fixation pathways in prokaryotes, warranting further study.

The intestinal microbiota relies heavily on digesting plant-derived polysaccharides into SCFAs for energy. SCFAs are crucial for intestinal health and as signaling molecules, influencing metabolism or peripheral tissue function by regulating gluconeogenesis and lipid accumulation (Carmody and Bisanz, 2023). The concentration of SCFAs depends on the intestinal microbiota composition, intestinal passage time, and the host diet’s fiber content. In the colon, acetic, propionic, and butyric acids account for approximately 90% of total SCFAs. Hu et al. reported that acetate promotes adipogenic differentiation in IM-BAT cells, increasing PPARγ and UCP1 mRNA expression, dependent on GPR43 activation by acetic acid (Hu et al., 2016). Yoshida et al. suggested that propionic acid might have preventive and therapeutic effects on diabetes mellitus by inhibiting gluconeogenesis. Supplementation with butyric acid increased energy expenditure and reduced obesity risk in high-fat diet-induced obese mice (Coppola et al., 2021).

However, in a clinical study, obese patients had a 20% higher fecal concentration of short-chain fatty acids (SCFAs) and higher levels of acetic, propionic, and butyric acids than lean patients (Schwiertz et al., 2010). This may be a compensatory protective mechanism against obesity, where more SCFAs are excreted from the feces to prevent increased accumulation of SCFAs in the intestinal, potentially leading to weight gain (Ríos-Covián et al., 2016; Blaak et al., 2020). Some studies have reported that acetic acid shows more obesogenic potential by acting as a substrate for lipogenesis and cholesterol synthesis. High-fat diets increase the absorption of LPS, which is associated with metabolic endotoxemia and induces inflammation leading to obesity. Large amounts of acetate in the gut are readily absorbed and travel through the portal circulation to the liver, where it is used to synthesize cholesterol. Researchers also found significant increases in norepinephrine, glucagon, and fatty acid binding protein 4 (FABP4) after consuming diets containing propionate, suggesting that propionate may be a “metabolic disruptor” that increases the risk of diabetes and obesity (Tirosh et al., 2019). In addition, high-fat diet decreases the amount of SCFAs in the intestinal, but a refined high-fat diet increases the amount of SCFAs in the intestinal. A refined high-fat diet increases gut microbiota biodiversity and SCFA content, thereby increasing energy metabolism (Wang et al., 2020). This finding may be due to the fact that a high-fat diet increases the production of intestinal acetate, which activates the parasympathetic nervous system to increase hunger hormones, as well as glucose-stimulated insulin secretion. This process creates a positive feedback loop from increased appetite, leading to food intake and weight gain, as well as fatty liver disease and insulin resistance (Perry et al., 2016). Nonetheless, this result is in contrast to previous studies that have shown that increased levels of SCFAs can lead to metabolic syndrome symptoms such as obesity.

In the present experiment, after feeding the rats high-fat diet, the total content of SCFAs in rat feces increased, while the content of caproic acid significantly decreased (*p* < 0.05). Compared to the MC group, the content of acetic acid, propionic acid, and the total content of SCFAs decreased in all intervention groups, suggesting that LGZGD promoted the metabolism of SCFAs by intestinal microorganisms. Notably, butyrate content was significantly higher in the Orlistat group, and hexanoic acid content was significantly lower in the LGZGD group. This result is consistent with previous studies on HFD diets, where Rau et al. found high levels of bacteria and SCFAs in the feces of NAFLD patients (Rau et al., 2018). Therefore, more sample analyses and clinical studies are needed to explain the relationship between obesity, intestinal microbiota, and SCFAs (Canfora et al., 2019).

Overall, correlation analysis found that *Christensenellaceae_R_7_group* and *UCG_005* positively correlated with obesity indicators, while *Muribaculaceaee* shows a positive correlation with Butyric acid and Isovaleric acid, which aligns with previous research findings (Wu et al., 2022; Wang et al., 2020; Zhang et al., 2019). Notably, *Muribaculaceae* are a family of bacteria within the order Bacteroidetes. *Muribaculaceae* produce SCFAs via endogenous mucin glycans and exogenous polysaccharides. Reports indicate that the increased abundance of *Muribaculaceae* is associated with the alleviative effects of a plant-based diet on conditions such as inflammatory bowel disease, obesity, and type 2 diabetes (Zhu et al., 2024; Qiao et al., 2024). This is consistent with the findings of our study. Therefore, this suggests that interventions from LGZGD can effectively reduce harmful intestinal microbiota, increase beneficial intestinal microbiota, and lower blood lipid levels, which may be the reason for their impact on intestinal microbiota functions. However, the exact mechanism needs to be confirmed in more clinical trials to meet clinical needs.

## 5 Conclusion

In summary, this study revealed that LGZGD moderated diversity, community structure, and functions of intestinal microbiota in obese rats fed with a high-fat diet. We hypothesize that the mechanism by which LGZGD anti-obesity may be due to its suppression of *Christensenellaceae_R_7_group*, *UCG_005*, caproic acid content, and promotion of *Muribaculaceaee*. This process improves hyperlipidemia, reduces the accumulation of fat in the body, and decreases the accumulation of lipid droplets in the liver. Therefore, LGZGD demonstrates the potential for preventing or treating obesity-related diseases. Next, we will delve deeper into how different dosages and durations of LGZGD administration affect its efficacy. We still need to conduct extensive laboratory and clinical experiments to validate these preliminary findings and to reveal the potential mechanisms of LGZGD anti-obesity action. This will provide more robust evidence to develop personalized treatments for obesity and related disorders.

## Data Availability

The datasets presented in this study can be found in online repositories. The names of the repository/repositories and accession number(s) can be found at: https://www.ncbi.nlm.nih.gov/, PRJNA1092620.

## References

[ref1] Ai Z. L. Zhang X. Ge W. Zhong Y. B. Wang H. Y. Zuo Z. Y.. (2022). *Salvia miltiorrhiza* extract may exert an anti-obesity effect in rats with high-fat diet-induced obesity by modulating gut microbiome and lipid metabolism. World J. Gastroenterol. 28, 6131–6156. doi: 10.3748/wjg.v28.i43.6131, PMID: 36483153 PMC9724488

[ref2] Bäckhed F. Ding H. Wang T. Hooper L. V. Koh G. Y. Nagy A.. (2004). The gut microbiota as an environmental factor that regulates fat storage. Proc. Natl. Acad. Sci. USA 101, 15718–15723. doi: 10.1073/pnas.0407076101, PMID: 15505215 PMC524219

[ref3] Blaak E. E. Canfora E. E. Theis S. Frost G. Groen A. K. Mithieux G.. (2020). Short chain fatty acids in human gut and metabolic health. Benefic. Microbes 11, 411–455. doi: 10.3920/BM2020.0057 32865024

[ref4] Bolyen E. Rideout J. R. Dillon M. R. Bokulich N. A. Abnet C. C. al-Ghalith G. A.. (2019). Reproducible, interactive, scalable and extensible microbiome data science using QIIME 2. Nat. Biotechnol. 37, 852–857. doi: 10.1038/s41587-019-0209-9, PMID: 31341288 PMC7015180

[ref5] Brown E. M. Clardy J. Xavier R. J. (2023). Gut microbiome lipid metabolism and its impact on host physiology. Cell Host Microbe 31, 173–186. doi: 10.1016/j.chom.2023.01.009, PMID: 36758518 PMC10124142

[ref6] Callahan B. J. McMurdie P. J. Rosen M. J. Han A. W. Johnson A. J. Holmes S. P. (2016). DADA2: high-resolution sample inference from Illumina amplicon data. Nat. Methods 13, 581–583. doi: 10.1038/nmeth.3869, PMID: 27214047 PMC4927377

[ref7] Canfora E. E. Meex R. Venema K. Blaak E. E. (2019). Gut microbial metabolites in obesity, NAFLD and T2DM. Nat. Rev. Endocrinol. 15, 261–273. doi: 10.1038/s41574-019-0156-z, PMID: 30670819

[ref8] Carmody R. N. Bisanz J. E. (2023). Roles of the gut microbiome in weight management. Nat. Rev. Microbiol. 21, 535–550. doi: 10.1038/s41579-023-00888-0 37138047 PMC13306846

[ref9] Chang C. J. Lin C. S. Lu C. C. Martel J. Ko Y. F. Ojcius D. M.. (2015). *Ganoderma lucidum* reduces obesity in mice by modulating the composition of the gut microbiota. Nat. Commun. 6:7489. doi: 10.1038/ncomms8489, PMID: 26102296 PMC4557287

[ref10] Coppola S. Avagliano C. Calignano A. Berni Canani R. (2021). The protective role of butyrate against Obesity and Obesity-related diseases. Molecules 26:682. doi: 10.3390/molecules26030682, PMID: 33525625 PMC7865491

[ref11] Cui H. Han S. Dai Y. Xie W. Zheng R. Sun Y.. (2023). Gut microbiota and integrative traditional Chinese and western medicine in prevention and treatment of heart failure. Phytomedicine 117:154885. doi: 10.1016/j.phymed.2023.154885, PMID: 37302262

[ref12] Dai L. Xu J. Liu B. Dang Y. Wang R. Zhuang L.. (2022). Lingguizhugan decoction, a Chinese herbal formula, improves insulin resistance in overweight/obese subjects with non-alcoholic fatty liver disease: a translational approach. Front. Med. 16, 745–759. doi: 10.1007/s11684-021-0880-3, PMID: 35471471

[ref13] Dang Y. Xu J. Yang Y. Li C. Zhang Q. Zhou W.. (2020). Ling-gui-zhu-Gan decoction alleviates hepatic steatosis through SOCS2 modification by N6-methyladenosine. Biomed. Pharmacother. 127:109976. doi: 10.1016/j.biopha.2020.109976 32559839

[ref14] de La Serre C. B. Ellis C. L. Lee J. Hartman A. L. Rutledge J. C. Raybould H. E. (2010). Propensity to high-fat diet-induced obesity in rats is associated with changes in the gut microbiota and gut inflammation. Am. J. Physiol. Gastrointest. Liver Physiol. 299, G440–G448. doi: 10.1152/ajpgi.00098.2010, PMID: 20508158 PMC2928532

[ref15] de Vadder F. Kovatcheva-Datchary P. Goncalves D. Vinera J. Zitoun C. Duchampt A.. (2014). Microbiota-generated metabolites promote metabolic benefits via gut-brain neural circuits. Cell 156, 84–96. doi: 10.1016/j.cell.2013.12.016, PMID: 24412651

[ref16] Dushay J. Chui P. C. Gopalakrishnan G. S. Varela–Rey M. Crawley M. Fisher F. M.. (2010). Increased fibroblast growth factor 21 in obesity and nonalcoholic fatty liver disease. Gastroenterology 139, 456–463. doi: 10.1053/j.gastro.2010.04.054, PMID: 20451522 PMC4862867

[ref17] Feng W. Yang Z. Liu Y. Chen R. Song Z. Pan G.. (2023). Gut microbiota: a new target of traditional Chinese medicine for insomnia. Biomed. Pharmacother. 160:114344. doi: 10.1016/j.biopha.2023.114344, PMID: 36738504

[ref18] GBD 2015 Obesity Collaborators Afshin A. Forouzanfar M. H. Reitsma M. B. Sur P. Estep K.. (2017). Health effects of overweight and Obesity in 195 countries over 25 years. N. Engl. J. Med. 377, 13–27. doi: 10.1056/NEJMoa1614362, PMID: 28604169 PMC5477817

[ref19] Geng J. Ni Q. Sun W. Li L. Feng X. (2022). The links between gut microbiota and obesity and obesity related diseases. Biomed. Pharmacother. 147:112678. doi: 10.1016/j.biopha.2022.112678 35134709

[ref20] Grice E. A. Segre J. A. (2011). The skin microbiome. Nat. Rev. Microbiol. 9, 244–253. doi: 10.1038/nrmicro2537, PMID: 21407241 PMC3535073

[ref21] Hsu Y. L. Chen C. C. Lin Y. T. Wu W. K. Chang L. C. Lai C. H.. (2019). Evaluation and optimization of sample handling methods for quantification of short-chain fatty acids in human Fecal samples by GC-MS. J. Proteome Res. 18, 1948–1957. doi: 10.1021/acs.jproteome.8b00536, PMID: 30895795

[ref22] Hu J. Kyrou I. Tan B. K. Dimitriadis G. K. Ramanjaneya M. Tripathi G.. (2016). Short-chain fatty acid acetate stimulates Adipogenesis and mitochondrial biogenesis via GPR43 in Brown adipocytes. Endocrinology 157, 1881–1894. doi: 10.1210/en.2015-1944, PMID: 26990063

[ref23] Huang W. Wang J. Kuang M. Xiao Z. Fan B. Sun G.. (2023). Exploring global research status and trends in anti-obesity effects of traditional Chinese medicine through intestinal microbiota: a bibliometric study. Front. Cell. Infect. Microbiol. 13:1271473. doi: 10.3389/fcimb.2023.1271473, PMID: 38045760 PMC10690589

[ref24] Huffman D. M. Barzilai N. (2009). Role of visceral adipose tissue in aging. Biochim. Biophys. Acta 1790, 1117–1123. doi: 10.1016/j.bbagen.2009.01.008, PMID: 19364483 PMC2779572

[ref25] Kleinert M. Clemmensen C. Hofmann S. M. Moore M. C. Renner S. Woods S. C.. (2018). Animal models of obesity and diabetes mellitus. Nat. Rev. Endocrinol. 14, 140–162. doi: 10.1038/nrendo.2017.161 29348476

[ref26] Lei Y. Tang L. Chen Q. Wu L. He W. Tu D.. (2022). Disulfiram ameliorates nonalcoholic steatohepatitis by modulating the gut microbiota and bile acid metabolism. Nat. Commun. 13:6862. doi: 10.1038/s41467-022-34671-1, PMID: 36369291 PMC9651870

[ref27] Ley R. E. Turnbaugh P. J. Klein S. Gordon J. I. (2006). Human gut microbes associated with obesity. Nature 444, 1022–1023. doi: 10.1038/4441022a 17183309

[ref28] Li Y. Yan Y. Fu H. Jin S. He S. Wang Z.. (2023). Does diet or macronutrients intake drive the structure and function of gut microbiota. Front. Microbiol. 14:1126189. doi: 10.3389/fmicb.2023.1126189, PMID: 36860485 PMC9970161

[ref29] Liu R. Hong J. Xu X. Feng Q. Zhang D. Gu Y.. (2017). Gut microbiome and serum metabolome alterations in obesity and after weight-loss intervention. Nat. Med. 23, 859–868. doi: 10.1038/nm.4358, PMID: 28628112

[ref30] Mocanu V. Zhang Z. Deehan E. C. Kao D. H. Hotte N. Karmali S.. (2021). Fecal microbial transplantation and fiber supplementation in patients with severe obesity and metabolic syndrome: a randomized double-blind, placebo-controlled phase 2 trial. Nat. Med. 27, 1272–1279. doi: 10.1038/s41591-021-01399-2, PMID: 34226737

[ref31] Perdomo C. M. Cohen R. V. Sumithran P. Clément K. Frühbeck G. (2023). Contemporary medical, device, and surgical therapies for obesity in adults. Lancet 401, 1116–1130. doi: 10.1016/S0140-6736(22)02403-5, PMID: 36774932

[ref32] Perry R. J. Peng L. Barry N. A. Cline G. W. Zhang D. Cardone R. L.. (2016). Acetate mediates a microbiome-brain-β-cell axis to promote metabolic syndrome. Nature 534, 213–217. doi: 10.1038/nature18309, PMID: 27279214 PMC4922538

[ref33] Petakh P. Oksenych V. Kamyshnyi A. (2023). The F/B ratio as a biomarker for inflammation in COVID-19 and T2D: impact of metformin. Biomed. Pharmacother. 163:114892. doi: 10.1016/j.biopha.2023.114892, PMID: 37196542 PMC10183625

[ref34] Qiao B. Xiao N. Deng N. Tan Z. (2024). Shenling Baizhu powder attenuates lard diet in a fatigued state-induced diarrhea via targeting microbial metabolites short chain fatty acids-mediated lipid metabolism. Biotech 14:203. doi: 10.1007/s13205-024-04045-z, PMID: 39157421 PMC11329475

[ref35] Rau M. Rehman A. Dittrich M. Groen A. K. Hermanns H. M. Seyfried F.. (2018). Fecal SCFAs and SCFA-producing bacteria in gut microbiome of human NAFLD as a putative link to systemic T-cell activation and advanced disease. United European Gastroenterol J 6, 1496–1507. doi: 10.1177/2050640618804444, PMID: 30574320 PMC6297934

[ref36] Redinger R. N. (2009). Fat storage and the biology of energy expenditure. Transl. Res. 154, 52–60. doi: 10.1016/j.trsl.2009.05.003 19595436

[ref37] Ríos-Covián D. Ruas-Madiedo P. Margolles A. Gueimonde M. de Los Reyes-Gavilán C. G. Salazar N. (2016). Intestinal short chain fatty acids and their link with diet and human health. Front. Microbiol. 7:185. doi: 10.3389/fmicb.2016.00185 26925050 PMC4756104

[ref38] Schwiertz A. Taras D. Schäfer K. Beijer S. Bos N. A. Donus C.. (2010). Microbiota and SCFA in lean and overweight healthy subjects. Obesity 18, 190–195. doi: 10.1038/oby.2009.167, PMID: 19498350

[ref39] Shi Q. Wang Y. Hao Q. Vandvik P. O. Guyatt G. Li J.. (2024). Pharmacotherapy for adults with overweight and obesity: a systematic review and network meta-analysis of randomised controlled trials. Lancet 403, e21–e31. doi: 10.1016/S0140-6736(24)00351-9, PMID: 38582569

[ref40] Suleiman J. B. Nna V. U. Zakaria Z. Othman Z. A. Bakar A. Usman U. Z.. (2020). Orlistat reverses intratesticular lactate transport decline and infertility in male obese rats. Reproduction 160, 863–872. doi: 10.1530/REP-20-0381, PMID: 33112813

[ref41] Tirosh A. Calay E. S. Tuncman G. Claiborn K. C. Inouye K. E. Eguchi K.. (2019). The short-chain fatty acid propionate increases glucagon and FABP4 production, impairing insulin action in mice and humans. Sci. Transl. Med. 11:eaav0120. doi: 10.1126/scitranslmed.aav0120, PMID: 31019023

[ref42] Wang B. Kong Q. Li X. Zhao J. Zhang H. Chen W.. (2020). A high-fat diet increases gut microbiota biodiversity and energy expenditure due to nutrient difference. Nutrients 12:3197. doi: 10.3390/nu12103197, PMID: 33092019 PMC7589760

[ref43] Wang Y. Yao W. Li B. Qian S. Wei B. Gong S.. (2020). Nuciferine modulates the gut microbiota and prevents obesity in high-fat diet-fed rats. Exp. Mol. Med. 52, 1959–1975. doi: 10.1038/s12276-020-00534-2, PMID: 33262480 PMC8080667

[ref44] Wang L. Zhou B. Zhao Z. Yang L. Zhang M. Jiang Y.. (2021). Body-mass index and obesity in urban and rural China: findings from consecutive nationally representative surveys during 2004-18. Lancet 398, 53–63. doi: 10.1016/S0140-6736(21)00798-4 34217401 PMC7617101

[ref45] Wu F. Lei H. Chen G. Chen C. Song Y. Cao Z.. (2022). Multiomics analyses reveal that long-term intake of Hesperetin-7-O-glucoside modulates the gut microbiota and bile acid metabolism in mice. J. Agric. Food Chem. 70, 14831–14840. doi: 10.1021/acs.jafc.2c05053, PMID: 36383360

[ref46] Xu J. Lian F. Zhao L. Zhao Y. Chen X. Zhang X.. (2015). Structural modulation of gut microbiota during alleviation of type 2 diabetes with a Chinese herbal formula. ISME J. 9, 552–562. doi: 10.1038/ismej.2014.177, PMID: 25279787 PMC4331591

[ref47] Ye X. Sun P. Lao S. Wen M. Zheng R. Lin Y.. (2023). Fgf21-Dubosiella axis mediates the protective effects of exercise against NAFLD development. Life Sci. 334:122231. doi: 10.1016/j.lfs.2023.122231 37935276

[ref48] Yue S. J. Wang W. X. Yu J. G. Chen Y. Y. Shi X. Q. Yan D.. (2019). Gut microbiota modulation with traditional Chinese medicine: a system biology-driven approach. Pharmacol. Res. 148:104453. doi: 10.1016/j.phrs.2019.104453, PMID: 31541688

[ref49] Zhang L. Ouyang Y. Li H. Shen L. Ni Y. Fang Q.. (2019). Metabolic phenotypes and the gut microbiota in response to dietary resistant starch type 2 in normal-weight subjects: a randomized crossover trial. Sci. Rep. 9:4736. doi: 10.1038/s41598-018-38216-9, PMID: 30894560 PMC6426958

[ref50] Zhang H. Y. Tian J. X. Lian F. M. Li M. Liu W. K. Zhen Z.. (2021). Therapeutic mechanisms of traditional Chinese medicine to improve metabolic diseases via the gut microbiota. Biomed. Pharmacother. 133:110857. doi: 10.1016/j.biopha.2020.110857 33197760

[ref51] Zhang S. Wang H. Zhu M. J. (2019). A sensitive GC/MS detection method for analyzing microbial metabolites short chain fatty acids in fecal and serum samples. Talanta 196, 249–254. doi: 10.1016/j.talanta.2018.12.049, PMID: 30683360

[ref52] Zhao L. (2013). The gut microbiota and obesity: from correlation to causality. Nat. Rev. Microbiol. 11, 639–647. doi: 10.1038/nrmicro3089 23912213

[ref53] Zhu Y. Chen B. Zhang X. Akbar M. T. Wu T. Zhang Y.. (2024). Exploration of the Muribaculaceae family in the gut microbiota: diversity, metabolism, and function. Nutrients 16:2660. doi: 10.3390/nu16162660, PMID: 39203797 PMC11356848

